# Strokes following snakebite envenomations: A systematic review and individual patient data meta-analysis

**DOI:** 10.1371/journal.pntd.0013789

**Published:** 2025-12-04

**Authors:** Thiago Almeida, Suelen Pereira Priante, Guilherme Pivoto João, Débora Nery Oliveira, Gabriel Mouta, Jacqueline Sachett, Luiz Carlos de Lima Ferreira, Robson Luís Oliveira de Amorim, Leandra Lobo, Marco Aurélio Sartim, Vanderson Sampaio, Fernando Almeida-Val, Wuelton Monteiro

**Affiliations:** 1 Escola Superior de Ciências da Saúde, Universidade do Estado do Amazonas, Manaus, Brazil; 2 Departamento de Pesquisa, Fundação de Medicina Tropical Dr Heitor Vieira Dourado, Manaus, Brazil; 3 Dipartamento di Biotecnologie Mediche, Università degli Studi di Siena, Siena, Italia; 4 Faculdade de Medicina, Universidade Federal do Amazonas, Manaus, Brazil; 5 Faculdade de Ciências Farmacêuticas, Universidade Federal do Amazonas, Manaus, Brazil; 6 Duke Global Health Institute, Duke University, Durham, North Carolina, United States of America; French Guiana University Hospital, FRENCH GUIANA

## Abstract

**Background:**

Snakebite envenomings (SBE) are an important and neglected health issue due to their frequency and potential for severe clinical outcomes. Envenomations can cause local and systemic complications, depending on the snake species, amount of venom injected, comorbidities, timing and use of antivenom, and access to health care. Systemic effects may be fatal or lead to permanent sequelae, including strokes resulting from venom-induced vascular and tissue damage. The objective of this study is to investigate the main clinical and epidemiological characteristics of individuals who developed stroke following SBE and to identify predictors of death.

**Methodology/principal findings:**

We conducted a systematic review and individual patient data meta-analysis using a predefined search strategy across MEDLINE/PubMed, LILACS, and SciELO databases, following PRISMA guidelines. A total of 100 studies were included, predominantly case reports and case series, comprising 130 individuals with stroke following SBE. Most patients were male (62.3%) and aged between 40 and 59 years (37.7%). Viperids caused 96.4% of the snakebites, particularly *Daboia russelii* and *Bothrops* spp. Most patients (90%) received antivenom therapy. Reported cases of snakebite-related stroke originated from 22 countries, mostly from India (36.9%), Brazil (13.9%) and Sri Lanka (10.8%). Ischemic strokes were more common than hemorrhagic strokes (61.5% vs. 38.5%), and multifocal brain involvement was predominant in both stroke types. Overall case-fatality was 23.4%. Sepsis [OR=6.21 (1.35-33.47); P = 0.001] and thrombocytopenia [OR=3.97 (1.66-10.03); P = 0.02] were predictors of deaths. Hemorrhagic stroke [OR=2.67 (1.15-6.31); P = 0.02], multiple brain lesions in a single hemisphere [OR=7.57 (2.33-33.39); P < 0.001], and subarachnoid hemorrhage [OR=7.00 (1.87-29.4)); P = 0.001] significantly increased the risk of death. Motor sequelae remained the most common long-term outcome (22.4%), occurring significantly more often in ischemic stroke survivors (28.8% vs. 9.4%, P = 0.05). Autopsy findings revealed intense brain alterations generally in parallel with damage in other organs such as the kidneys, lung, and heart.

**Conclusions/significance:**

Strokes from SBE represent a potential medical emergency in low- and middle-income countries where snakebites predominate, and lead to high rates of mortality and long-term disability. Recognizing stroke as a disabling and underreported consequence of snakebite is essential for improving clinical outcomes and guiding public health responses. Integrating the knowledge on predictors of death from SBE-relate strokes into health policies will be vital for reducing long-term morbidity and advancing disability-inclusive strategies.

## Author summary

Snakebites are a major public health problem in tropical regions, where people often work in farming, fishing, or forest activities. While many snakebites cause local pain and swelling, some venoms can also damage blood vessels and blood clotting, leading to severe complications. One of the most serious but less recognized outcomes is stroke, which can cause death or life-long disability. In this study, we reviewed published reports of 130 people who developed stroke after a snakebite. Most of them were men in working age, and nearly all bites were caused by vipers, especially Russell’s viper and lancehead snakes. Strokes happened quickly in many patients, often within the first day, but sometimes days later. Both ischemic strokes (caused by blocked blood flow) and hemorrhagic strokes (caused by bleeding) were observed, with many affecting several parts of the brain at once. About one in four patients died, and many survivors were left with motor, speech, or vision problems that limit daily life. Our findings show that stroke after snakebite is more common and severe than usually recognized. Improving awareness, early treatment, and access to rehabilitation is essential to reduce the burden of death and disability from snakebite envenoming.

## Introduction

Snakebites envenomings (SBE) commonly affect people living in intertropical regions and greatly impact regional socioeconomic development [1]. It is estimated that 5.4 million SBEs and 138,000 deaths occur annually, in addition to the progressive increase in permanent sequelae [2]. SBEs are the result of the inoculation of snake venom and the clinical consequences depend on factors such as the species involved, the amount of toxins injected, access to proper healthcare, and the individuals’ health status [1,3]. The enormous interspecies and intraspecies variability of snake venoms translates in a wide range of clinical manifestations, from local effects at the bite site to severe systemic complications, which are significantly more severe in rural areas of low-resource settings with poor access to antivenoms [4]. Local signs include pain, swelling and bruising, which in severe cases can progress to blistering, secondary bacterial infection, tissue necrosis and compartment syndrome [5–7]. Systemic effects can range from headache, nausea and vomiting to neuromuscular paralysis [8], bleeding [9,10] and acute kidney injury [11,12]. Extreme age groups may experience more severe envenoming due to their smaller size and higher venom-to-body weight ratio in children [13] and higher frequency of chronic comorbidities in the elderly [14]. Respiratory failure, systemic bleeding, sepsis, shock and brain stroke were recorded only among fatal cases [15,16].

Venoms from both viperid and elapid snakes include digestive hydrolases, phospholipases, thrombin-like pro-coagulant, serine proteases and metalloproteases [17,18]. Once injected the body, the toxins cause injury on surrounding tissues, and spread systemically through the lymphatic system and bloodstream, affecting multiple organs [3]. These components of the snake venom can affect hemostasis by activating or inhibiting coagulant factors or platelets, disrupting endothelium or causing thrombosis [10,19]. Complications range from unclottable blood with no clinical relevance or minor systemic bleeding (spontaneous bleeding from recent skin injuries, conjunctival bleeding, gingival bleeding, hematuria among others) [9] to life-threatening cases of large vessels’ ischemia [20,21], myocardial infarction [22,23], ischemic or hemorrhagic brain stroke [16], and circulatory shock [24].

Acute stroke is the acute onset of focal neurological deficits in a vascular territory affecting the brain, retina, or spinal cord due to underlying cerebrovascular diseases [25]. Strokes are classified as ischemic and hemorrhagic, and hemorrhagic strokes can further be classified as intracerebral and subarachnoid hemorrhage [26]. Ischemic stroke, blocking blood flow to the brain, represents about 87% of all strokes, and is a leading cause of disability and mortality worldwide [27]. Hemorrhagic stroke is due to bleeding into the brain by the rupture of a blood vessel [28]. Although hypertension, lack of physical activity, abnormal lipids, unhealthy diet, abdominal obesity, psychological factors, current smoking, cardiac causes, alcohol consumption and diabetes account for about 90% risk of stroke in low and middle-income countries [29], infectious diseases (tuberculosis, syphilis, HIV/Aids, malaria, Chagas’ disease, rheumatic heart disease, infective endocarditis, mycotic aneurysms), sickle cell disease and snakebites also contribute to this burden [30].

Cerebrovascular events following SBE, such as intracerebral or subarachnoid hemorrhages and ischemic strokes, are linked to high morbidity and mortality, and survivors are often left with disabling neurological sequelae [16]. Likewise, effective treatment of SBE complications such as stroke need prompt diagnosis and emergency neurological life support to maximize recovery, which are often inequitable in low- and middle-income countries [30,31].

In this study, we systematically review the literature on strokes following SBE to characterize the clinical and epidemiological profiles of affected individuals and to identify predictors of death from a meta-analyses of individual participant data.

## Methods

### Literature review

We systematically searched the MEDLINE (via PubMed), Web of Science, and LILACS databases for reports of stroke secondary to snakebite envenomation. The search was conducted in accordance with the Preferred Reporting Items for Systematic Reviews and Meta-Analyses of Individual Participant Data (PRISMA-IPD) guidelines [32; S1 File]. The review was not registered. Eligible studies included case reports and case series describing the clinical and epidemiological features of stroke following snakebite envenomation, regardless of language, publication year, or geographic location.

Three independent reviewers (TA, SPP, and WM) performed the screening and data extraction. Discrepancies were resolved through discussion and consensus. The search strategy used a combination of controlled vocabulary and free-text terms, including but not limited to: (Snakebite OR Snake Bite OR Snake Envenomation OR Snakebite Envenomation OR Snake Envenoming OR Snakebite Envenoming) AND (Stroke OR Cerebrovascular Accident OR Brain Vascular Accident OR Cerebrovascular Stroke OR Cerebral Stroke OR Brain Ischemia OR Ischemic Encephalopathy OR Cerebral Ischemia OR Brain Hemorrhage OR Hemorrhagic Encephalopathy OR Cerebral Hemorrhage).

Initial screening was based on titles and abstracts. When stroke diagnosis was unclear, the full text was reviewed to confirm eligibility. Additional references were identified through backward citation tracking of included articles, relevant reviews, opinion pieces, and textbooks. The final search was completed on January 30, 2025.

### Study measures

The primary outcome was the occurrence of ischemic, hemorrhagic, or mixed-type strokes secondary to snakebite envenomation. For each case, stroke injuries were classified as either isolated or multiple, and the affected brain regions were categorized according to vascular territories: middle cerebral artery (MCA), anterior cerebral artery (ACA), posterior cerebral artery (PCA), and basilar artery.

Information on neurological complications during hospitalization - specifically motor and sensory impairments - and long-term disabilities was also collected. Clinical outcomes were categorized as recovery without complaints, presence of sequelae, or death.

Additional data were extracted to describe demographic, clinical, laboratory, and pathological characteristics. These included: patient sex, age, country of occurrence, use of traditional or popular treatments, time from bite to medical assistance, anatomical site of the bite, local complications (e.g., necrosis, secondary infection, compartment syndrome), systemic complications (e.g., acute kidney injury, bleeding), disorders of primary hemostasis (e.g., thrombocytopenia), disorders of secondary hemostasis (e.g., venom-induced coagulopathy), presence of comorbidities, snake species involved, duration of hospitalization, antivenom administration, and early adverse reactions to antivenom.

### Statistical analyses

Descriptive statistics were used to summarize the demographic, epidemiological, and clinical characteristics of patients with stroke secondary to snakebite envenomation. Categorical variables were reported as frequencies and percentages, while continuous variables were expressed as means and standard deviations or medians and interquartile ranges, as appropriate. Comparisons between ischemic and hemorrhagic stroke groups were performed using the chi-squared test or Fisher’s exact test, when necessary. Risk factors associated with death and long-term disabilities were assessed using odds ratios (ORs) and corresponding 95% confidence intervals (95% CI), based on relevant demographic, clinical, and epidemiological variables. Univariate logistic regression models to estimate ORs. A two-tailed P-value of <0.05 was considered statistically significant.

## Results

### Patients’ characteristics

**Fig 1:**
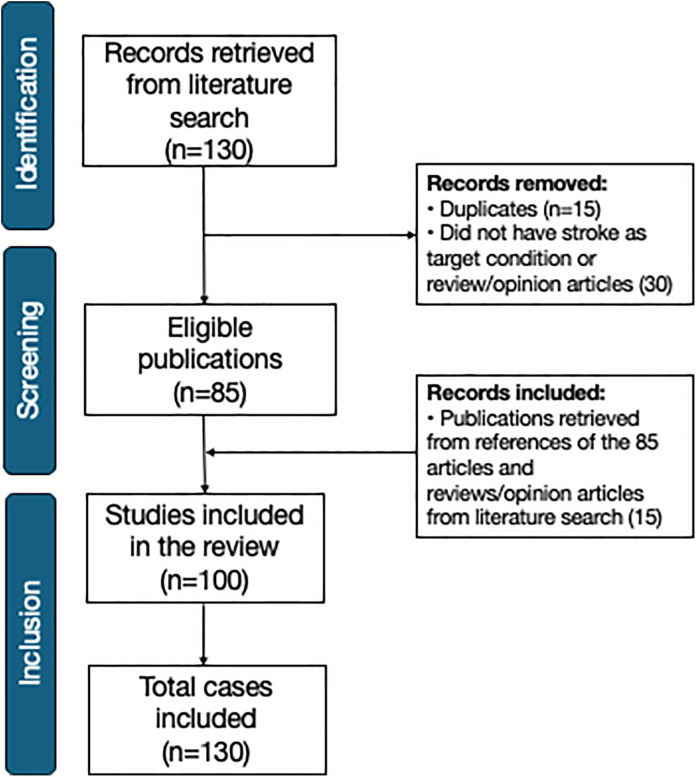
PRISMA flow diagram of study selection for the systematic review.

A total of 130 individual cases of stroke secondary to snakebite envenomation were included in this review, retrieved from 101 publications [33–133] (Fig 1). Strokes were diagnosed by computed tomography (CT; 89 cases, 68.5%), magnetic resonance imaging (MRI; 21 cases, 16.2%), CT plus MRI (10 cases, 7.7%), and autopsy (6 cases, 4.6%). Four cases (3%) had no information on the diagnosis method. As shown in Table 1, most patients were male (61.5%) and aged between 40 and 59 years (37.7%). Lower limbs were the most frequent anatomical site of the bite (77.5%), and in most cases (96.4%), the envenomation was caused by snakes from the Viperidae family. Patients who developed hemorrhagic stroke were significantly more likely to have experienced a delay of more than 24 hours between the bite and initial care (28% vs. 11.1%; P = 0.01). Local complications such as secondary infection (13.1%) and necrosis (5.4%) were similarly distributed among all ischemic and hemorrhagic cases. The most prevalent systemic complications were systemic bleeding (26.9%) and acute kidney injury (15.4%). Headache was significantly more frequent in patients with hemorrhagic stroke (14% vs. 2.5%; P = 0.03). Most patients (90%) received antivenom therapy, with early adverse reactions reported in 5.4% of cases.

**Table 1:** Demographic, clinical, and envenomation-related characteristics of patients with stroke following snakebite envenomations.

Variables	Total (n = 130)	Ischemic (n = 80)	Hemorrhagic (n = 50)	*P*
**Male (n, %) (n = 130)**	80 (61.5%)	50 (62.5%)	30 (60%)	0.67
**Age groups (n, %) (n = 130)**				
0-9	4 (3.1%)	3 (3.8%)	1 (2%)	
10-19	19 (14.6%)	8 (10%)	11 (22%)	0.83
20-39	33 (25.4%)	24 (30%)	9 (18%)	0.91
40-59	49 (37.7%)	35 (43.7%)	14 (28%)	0.94
≥60	25 (19.2%)	10 (12.5%)	15 (30%)	0.73
**Time from bite to medical assistance (n = 102; hours)**				
<1	20 (19.6%)	15 (23.8%)	5 (10%)	
1-6	33 (32.3%)	24 (38.1%)	9 (18%)	0.85
7-24	28 (27.5%)	17 (27%)	11 (22%)	0.30
>24	21 (20.6%)	7 (11.1%)	14 (28%)	0.01
**Anatomical region of the bite (n = 111)**				
Upper limbs	25 (22.5%)	12 (18.5%)	13 (26%)	
Lower limbs	86 (77.5%)	53 (81.5%)	33 (66%)	0.22
**Snake family (n = 111)**				
Elapidae	4 (3.6%)	2 (2.7%)	2 (4%)	
Viperidae	107 (96.4%)	73 (97.3%)	35 (70%)	0.80
**Local manifestations/complications (n = 130)**				
Secondary infection	17 (13.1%)	10 (12.5%)	7 (14%)	>0.99
Necrosis	7 (5.4%)	3 (3.8%)	4 (8%)	0.51
Compartment syndrome	4 (3.1%)	4 (5%)	0 (0%)	0.28
**Systemic manifestations/complications (n = 130)**				
Systemic bleeding	35 (26.9%)	17 (21.3%)	18 (36%)	0.06
Acute kidney injury	20 (15.4%)	13 (16.3%)	7 (14%)	0.73
Nausea and/or vomiting	15 (11.5%)	6 (7.5%)	9 (18%)	0.07
Seizure	10 (7.7%)	5 (6.3%)	5 (10%)	0.43
Headache	9 (6.9%)	2 (2.5%)	7 (14%)	0.03
Sepsis	8 (6.2%)	4 (5%)	4 (8%)	0.73
Rhabdomyolysis	2 (1.5%)	2 (2.5%)	0 (0%)	0.75
Myocarditis	2 (1.5%)	2 (2.5%)	0 (0%)	0.75
Deep vein thrombosis	2 (1.5%)	2 (2.5%)	0 (0%)	0.75
Endocarditis	1 (0.8%)	0 (0%)	1 (2%)	0.77
Acute myocardial infarction	1 (0.8%)	1 (1.3%)	0 (0%)	>0.99
Pleural effusion	1 (0.8%)	0 (0%)	1 (2%)	0.77
Pericardial effusion	1 (0.8%)	0 (0%)	1 (2%)	0.77
Pulmonary edema	1 (0.8%)	1 (1.3%)	0 (0%)	>0.99
Aortic embolism	1 (0.8%)	0 (0%)	1 (2%)	0.77
Pulmonary embolism	1 (0.8%)	0 (0%)	1 (2%)	0.77
Carotid artery occlusion	1 (0.8%)	1 (1.3%)	0 (0%)	>0.99
**Hemostasis disorders (n = 130)**				
Thrombocytopenia^1^	59 (45.4%)	36 (45%)	23 (46%)	0.91
Venom-induced consumption coagulopathy^2^	94 (72.3%)	62 (77.5%)	32 (64%)	0.09
**Presence of comorbidities**^**3**^ **(n = 130)**	10 (7.7%)	5 (6.3%)	5 (10%)	0.43
**Time of hospital stay (n = 85; weeks)**				
1	10 (11.8%)	3 (6.5%)	7 (14%)	
2-4	12 (14.1%)	9 (19.6%)	3 (6%)	0.09
4-8	20 (23.5%)	11 (23.9%)	9 (18%)	0.36
8-15	26 (30.6%)	16 (34.8%)	10 (20%)	0.18
15-20	9 (10.6%)	4 (8.7%)	5 (10%)	0.86
20-30	2 (2.4%)	1 (2.2%)	1 (2%)	>0.99
>30	6 (7%)	2 (4.3%)	4 (8%)	>0.99
**Antivenom use (n = 130)**	117 (90%)	69 (86.3%)	48 (96%)	0.12
**Early adverse reactions to AV (n = 117)**	7 (5.4%)	4 (3.1%)	3 (6%)	>0.99
**Traditional medicine (n = 130)**	10 (5.4%)	4 (3.1%)	6 (12%)	0.26

^1^Defined as platelet counts <150,000/mm^3^.

^2^Defined as incoagulable blood in clotting tests, abnormal elevation of International Normalized Ratio (INR), and hypofibrinogenemia (<150 mg/dl).

^3^Six patients with hypertension, three with coronary artery disease and one with diabetes.

Reported cases of snakebite-related stroke originated from 22 countries. The majority were reported in India, which accounted for 48 cases (36.9%) of the total. Other countries with notable case frequencies included Brazil with 18 cases (13.9%), Sri Lanka with 14 cases (10.8%), and Morocco with 9 cases (6.9%). The remaining cases were distributed among Thailand (5 cases, 3.8%), Iran (4 cases, 3.1%), Nepal (3 cases, 2.3%), Turkey (3 cases, 2.3%), and the United States, China, Colombia, Nigeria, South Africa, Bangladesh, Pakistan, Malaysia, Laos, Taiwan, Vietnam, Indonesia, Papua New Guinea, and Ecuador, each contributing with one or two cases (≤1.5%).

Most reports involved envenomation by Viperidae species, particularly *Daboia russelii* and *Bothrops* spp. Among ischemic cases, the majority were caused by snakes from the Viperidae family, including *Trimeresurus stejnegeri*, *Deinagkistrodon acutus*, *Daboia russelii*, *Bothrops lanceolatus*, *Crotalus oreganus helleri*, *Cerastes cerastes*, *Echis carinatus*, *Hypnale hypnale*, *Agkistrodon blomhoffii brevicaudus*, *Crotalus viridis viridis*, *Calloselasma rhodostoma*, and *Trimeresurus gramineus*. A single Elapidae species, *Pseudonaja textilis*, was also associated with ischemic events. Hemorrhagic strokes were predominantly linked to Viperidae species such as *Bothrops atrox*, *Daboia russelii*, *Bothrops jararacussu*, *Bothrops marajoensis*, *Bothrops asper*, *Cerastes cerastes*, and *Echis ocellatus*. Additionally, two Elapidae species - *Notechis scutatus* and *Pseudonaja textilis* - were implicated in hemorrhagic events. This distribution highlights a predominance of Viperidae envenomation in both ischemic and hemorrhagic presentations, although Elapidae-related strokes were also observed.

Individual characteristics of the 130 patients are detailed in S2 File.

### Stroke characteristics

Stroke characteristics are summarized in Table 2. Ischemic strokes were more common than hemorrhagic strokes (61.5% vs. 38.5%). In both stroke types, multifocal brain involvement was predominant: 60.8% of cases presented with multiple infarcts or hemorrhages in the same hemisphere. The middle cerebral artery (MCA) territory was the most frequently affected region in ischemic strokes (23.1%), followed by the anterior cerebral artery (ACA), posterior cerebral artery (PCA), and basilar artery. Among patients with hemorrhagic stroke, 61.9% had multiple hemorrhagic foci. Conservative management was adopted in 93.1% of cases, while 6.9% underwent surgical intervention. The mean Glasgow Coma Scale (GCS) score at admission was lower among patients with hemorrhagic stroke (8.0) than those with ischemic stroke (9.4).

**Table 2:** Neuroimaging findings and stroke characteristics by stroke type (ischemic vs. hemorrhagic).

Variable, n (%)	Total	Ischemic	Hemorrhagic
**Stroke injury (n = 125)**			
Isolated	49 (39.2%)	33 (41.8%)	16 (34.8%)
Multiple in a single hemisphere	76 (60.8%)	46 (58.2%)	30 (65.2%)
**Site of brain injury (n = 117)**			
Multiple infarcts	42 (35.9%)	42 (56%)	...
Middle cerebral artery	27 (23.1%)	20 (26.7%)	7 (16.7%)
Multiple hemorrhages	26 (22.2%)	...	26 (61.9%)
Anterior cerebral artery	8 (6.8%)	2 (2.7%)	6 (14.3%)
Posterior cerebral artery	7 (6%)	6 (8%)	1 (2.4%)
Basilar artery	7 (6%)	5 (6.6%)	2 (4.7%)
**Type of treatment (n = 130)**			
Conservative	121 (93.1%)	77 (96.3%)	44 (88%)
Surgical	9 (6.9%)	3 (3.7%)	6 (12%)
Glasgow Coma Scale at admission (Mean ± SD)	8.8 (3.5)	9.4 (3.8)	8.0 (3.0)

### Predictors of case-fatality

Table 3 summarizes clinical variables associated with mortality. Among patients who died (n = 30/128; 23.4%), thrombocytopenia was significantly more frequent compared to survivors (70.0% vs. 36.7%, P = 0.001). Sepsis was also more common among fatal cases (16.7% vs. 3.1%, P = 0.03). Time of hospital stay was also significantly different between groups (P < 0.05). Other clinical complications such as secondary infection, necrosis, and compartment syndrome showed no significant differences between groups. The use of antivenom and traditional medicine, as well as early adverse reactions, were not associated with mortality.

**Table 3:** Predictors of death from stroke caused by snakebite envenomation.

Variable, n (%)	Survival	Death	OR (95%CI)	*P*
**Gender (Female)**	38 (38.8%)	11 (36.7%)	0.98 (0.38-2.13)	0.84
**Age groups (years) (n = 128)**				
0-9	2 (2%)	2 (6.7%)		
10-19	13 (13.2%)	5 (16.7%)	0.37 (0.03-4.42)	0.70
20-39	27 (27.6%)	6 (20%)	0.23 (0.02-2.63)	0.39
40-59	35 (35.8%)	13 (43.3%)	0.37 (0.03-3.85)	0.63
≥60	21 (21.4%)	4 (13.3%)	0.21 (0.02-2.46)	0.36
**Time from bite to medical assistance (hours) (n = 110)**				
<1	13 (17.3%)	7 (28%)		
1-6	26 (34.7%)	5 (20%)	0.36 (0.08-1.40)	0.12
7-24	21 (28%)	7 (28%)	0.62 (0.17-2.27)	0.45
>24	15 (20%)	6 (24%)	0.74 (0.19-2.89)	0.65
**Anatomical region of the bite (n = 110)**				
Upper limbs	17 (20.2%)	8 (30.8%)		
Lower limbs	67 (79.8%)	18 (69.2%)	0.57 (0.21-1.61)	0.26
**Snake family (n = 111)**				
Elapidae	1 (1.2%)	3 (10.7%)		
Viperidae	82 (98.8%)	25 (89.3%)	0.10 (0.01-1.02)	0.09
**Local manifestations/complications (n = 128)**				
Secondary infection	10 (10.2%)	7 (23.3%)	2.65 (0.87-7.85)	0.06
Necrosis	5 (5.1%)	2 (6.7%)	1.33 (0.17-7.11)	>0.99
Compartment syndrome	2 (2%)	2 (6.7%)	3.39 (0.34-33.76)	0.47
**Systemic manifestations/complications (n = 128)**				
Systemic bleeding	26 (26.5%)	9 (30%)	1.18 (0.46-2.90)	0.71
Acute kidney injury	12 (12.2%)	8 (26.7%)	2.28 (0.79-6.36)	0.10
Nausea and/or vomiting	8 (8.2%)	7 (23.3%)	3.38 (1.06-10.61)	0.06
Sepsis	3 (3.1%)	5 (16.7%)	6.21 (1.35-33.47)	0.03
Headache	9 (9.2%)	0 (0%)	...	0.16
Seizure	7 (7.1%)	3 (10%)	1.44 (0.29-5.88)	0.85
Rhabdomyolysis	2 (2%)	0 (0%)	...	>0.99
Myocarditis	2 (2%)	0 (0%)	...	>0.99
Endocarditis	0 (0%)	1 (3.3%)	...	0.46
Acute myocardial infarction	0 (0%)	1 (3.3%)	...	0.46
Pleural effusion	0 (0%)	1 (3.3%)	...	0.46
Pericardial effusion	1 (1%)	0 (0%)	...	>0.99
Pulmonary edema	0 (0%)	1 (3.3%)	...	0.46
Deep vein thrombosis	2 (2%)	0 (0%)	...	>0.99
Aortic embolism	1 (1%)	0 (0%)	...	>0.99
Pulmonary embolism	1 (1%)	0 (0%)	...	>0.99
Carotid artery occlusion	1 (1%)	0 (0%)	...	>0.99
**Hemostasis disorders (n = 128)**				
Thrombocytopenia^1^	36 (36.7%)	21 (70%)	3.97 (1.66-10.03)	0.001
Venom-induced consumption coagulopathy^2^	71 (72.4%)	21 (70%)	0.89 (0.36-2.27)	0.79
**Presence of comorbidities** ^**3**^	7 (7.2%)	3 (10%)	1.44 (0.28-5.89)	0.86
**Time of hospital stay (n = 85; weeks)**				
1	2 (3.2%)	8 (36.4%)		
2-4	9 (14.3%)	3 (13.6%)	10.4 (1.49-107.7)	0.03
4-8	13 (20.6%)	7 (31.8%)	6.91 (1.21-58.93)	0.05
8-15	24 (38.1%)	2 (9.1%)	38.86 (5.48-438.5)	<0.001
>15	15 (23.8%)	2 (9.1%)	24.46 (3.34-282.4)	<0.001
**Antivenom use (n = 128)**	87 (88.7%)	28 (93.3%)	1.76 (0.41-12.33)	0.74
**Early adverse reactions to antivenom (n = 128)**	5 (5.7%)	2 (7.1%)	0.79 (0.15-6.21)	>0.99
**Traditional medicine (n = 128)**	7 (7.2%)	3 (10%)	1.44 (0.28-5.89)	0.86

^1^Defined as platelet counts <150,000/mm^3^.

^2^Defined as incoagulable blood in clotting tests, abnormal elevation of International Normalized Ratio (INR), and hypofibrinogenemia (<150 mg/dl).

^3^Six patients with hypertension, three with coronary artery disease and one with diabetes.

Stroke-related variables were also associated with mortality (Table 4). Hemorrhagic stroke, multiple brain lesions in a single hemisphere, and subarachnoid hemorrhage significantly increased the risk of death.

**Table 4:** Stroke characteristics and risk of death.

Variable, n (%)	Survival (n = 98)	Death (n = 30)	OR (95%CI)	*P*
**Stroke type (n = 128)**				
Ischemic	66 (67.3%)	13 (43.3%)		
Hemorrhagic	32 (32.7%)	17 (56.7%)	2.67 (1.15-6.31)	0.02
**Stroke injury (n = 126)**				
Isolated	47 (48%)	3 (10%)		
Multiple in a single hemisphere	51 (52%)	25 (90%)	7.57 (2.33-33.39)	<0.001
**Site of brain injury (n = 117)**				
Multiple infarcts	32 (34%)	10 (43.5%)		
Multiple hemorrhages	16 (17%)	10 (43.5%)	1.98 (0.67-5.89)	0.31
Middle cerebral artery	27 (28.7%)	0 (0%)	0.12 (0.01-0.79)	0.01
Anterior cerebral artery	7 (7.4%)	1 (4.3%)	0.46 (0.02-3.53)	0.86
Posterior cerebral artery	6 (6.4%)	1 (4.3%)	0.54 (0.02-4.27)	>0.99
Basilar artery	6 (6.4%)	1 (4.3%)	0.54 (0.02-4.27)	>0.99
**Subarachnoid hemorrhage**	4 (4.1%)	7 (23.3%)	7.00 (1.87-29.4)	0.01
**Cerebral edema**	18 (18.4%)	6 (20%)	1.11 (0.37-3.06)	>0.99
**Brain herniation**	8 (8.2%)	4 (13.3%)	1.72 (0.42-6.18)	0.59
**Type of treatment (n = 128)**				
Conservative	90 (91.8%)	29 (96.7%)		
Surgical	8 (8.2%)	1 (3.3%)	0.39 (0.02-2.59)	0.66
**Glasgow Coma Scale at admission**	9.4 (3.5)	7.5 (3.3)		0.08

### Disabilities from SBE-related strokes

Disabilities associated with stroke following SBE are presented in Table 5. During hospitalization, motor complications were the most frequent (63.1%), followed by visual (22.3%), speech (20.0%), and sensory complications (12.3%), with no significant differences between ischemic and hemorrhagic strokes. Among the 98 patients with follow-up data, motor sequelae remained the most common long-term outcome (22.4%), occurring significantly more often in ischemic stroke survivors (28.8% vs. 9.4%, P = 0.05). Speech and visual sequelae were observed in 11.2% and 9.2% of cases, respectively, while sensory sequelae and unspecified impairments were less frequent. Follow-up duration ranged from 1 to 18 months.

**Table 5:** Complications during hospitalization and long-term sequelae among survivors.

Time of assessment	Total	Ischemic	Hemorrhagic	*P*
**In-hospital complications (n = 130)**				
Sensory complication	16 (12.3%)	10 (12.5%)	6 (12%)	>0.99
Visual complication	29 (22.3%)	21 (26.3%)	8 (16%)	0.17
Speech complication	26 (20%)	18 (22.5%)	8 (16%)	0.37
Motor complication	82 (63.1%)	55 (68.8%)	27 (54%)	0.09
**Post-discharge sequelae**^**1**^ **(n = 98)**				
Unspecified sequelae	5 (5.1%)	3 (4.5%)	2 (6.3%)	>0.99
Sensory sequela	2 (2%)	2 (3%)	0 (0%)	0.90
Visual sequela	9 (9.2%)	6 (9.1%)	3 (9.4%)	>0.99
Speech sequela	11 (11.2%)	10 (15.2%)	1 (3.1%)	0.14
Motor sequela	22 (22.4%)	19 (28.8%)	3 (9.4%)	0.05

^1^Follow-up duration ranged from 1 to 18 months.

Autopsy findings were reported in six fatal cases of stroke following snakebite envenomation, revealing a range of central and systemic pathological alterations. Cerebral hemorrhages were common, including diffuse subarachnoid and large-volume intracerebral hemorrhages, notably in the frontoparietal-temporal lobes and ventricles. In some cases, infarcts were multifocal and associated with embolic phenomena, as observed in both the brain and other organs such as the kidneys, spleen, and heart. Acute tubular necrosis, petechial renal hemorrhages, and cortical-medullary disorganization were consistent renal findings. Non-bacterial thrombotic endocarditis and vegetations on aortic valves were noted in one patient. Additional systemic findings included pulmonary and gastric hemorrhages, hepatosplenic petechiae, and signs of sepsis or multi-organ dysfunction. The detection of venom in brain tissue was reported in one case, further supporting a direct pathogenic role of venom components in cerebrovascular injury (Table 6).

**Table 6:** Autopsy findings in fatal cases of stroke following snakebite envenomation.

Patient (age and sex)	Snake	Clinical findings	Stroke type	Pathological findings	Reference
11/M	*Notechis scutatus*	Nausea, vomiting, abdominal pain, muscle weakness, and drowsiness. Pallor, blood pressure: 110/70 mmHg, pulse: 80 bpm. Progressed to irritability, aggressiveness, and loss of motor responses 26 hours after envenomation. Brainstem reflexes absent after 26 hours.	Hemorrhagic	Multiple intracerebral hemorrhages. Subarachnoid blood collections. No thrombi in intracerebral vessels. No evidence of cerebral infarction.	[36]
23/M	Viperid	Diarrhea and vomiting (lasted one day after the bite). Swelling of the foot and leg. Oliguria followed by anuria (beginning on the third day after the bite).	Ischemic	Heart: Presence of friable vegetations (0.5 to 0.8 cm) on the non-coronary and left coronary cusps of the aortic valve, composed of fibrin and platelets with mild inflammatory infiltrate. No organisms detected on stains or cultures, consistent with non-bacterial thrombotic endocarditis. Kidneys: Hydronephrotic left kidney with impacted calculus in the ureteropelvic junction; histology showed acute tubular necrosis with hemolysis, hemosiderin deposition, and degenerated tubules. Brain: Recent embolic infarcts, fibrin thrombi in capillaries, perivascular hemorrhages, and necrosis predominantly in the cerebral white matter. Other organs: Embolic infarcts identified in the kidneys, spleen, and brain.	[38]
50/M	*Crotalus oreganus helleri*	Pain, swelling, and tenderness to palpation involving approximately half of the left lower limb. Reported dyspnea. Paresthesia around the mouth and in both upper and lower limbs, with worsening symptoms in the left lower limb.	Ischemic	Significant multifocal infarction in the central nervous system. Multiple emboli in small vessels of the left ventricle, cardiac septum, and right lung, with areas of ischemia and congestion associated with a shower embolism pattern.	[70]
59/F	*Bothrops atrox*	Sweating, cold skin, tachycardia, sialorrhea, aphasia, blood pressure: 230/130 mmHg, and ECG: 3 points. Coma, midriatic pupils with no light response, hypothermia, bradycardia, anuria, and mechanical ventilation. Persistent hypothermia, bradycardia, and hypotension, progressing to cardiopulmonary arrest and death.	Hemorrhagic	Subarachnoid and intraparenchymal hemorrhage. Localization of *Bothrops* sp. venom in brain tissue.	[88]
53/M	*Daboia russelii*	Nausea, vomiting, abdominal pain, hemoptysis, and anuria for 12 hours. Gingival bleeding, bilateral ptosis, and swelling of the right lower limb. Blood pressure: 160/90 mmHg, heart rate: 72 bpm, respiratory rate: 22/min. WBCT20 test: more than 20 minutes.	Hemorrhagic	Lesion area identified in the parietal lobes of the brain. Reduced corticomedullary differentiation in both kidneys. Presence of petechial hemorrhages on the outer surface of the kidneys.	[90]
42/M	Viperid	Initial symptoms: Pain and mild swelling in the left foot. Progression after 4 hours: Altered mental status, nausea, vomiting, increased thirst, dizziness, chest pain, and sweating. Upon admission: Drowsiness and altered consciousness. Glasgow Coma Scale: 14. Blood pressure: 160/90 mmHg. Respiratory rate: 30/min.Pulse: 136 bpm. SpO₂: 98% on room air. Gross hematuria following catheterization.	Hemorrhagic	1. External examination: Fang marks (2 punctures) on the left foot, with edema, purplish discoloration, and bleeding upon pressure. Ecchymoses in the underlying layers of skin and muscles at the bite site. 2. Brain: Cerebral edema with diffuse subarachnoid hemorrhage. Intracerebral hemorrhage of 200 mL in the bilateral frontoparietal-temporal lobes. Hemorrhage of 50 mL in the third and fourth ventricles. 3. Abdominal cavity: Multiple petechial hemorrhages on the peritoneum and subcapsular surface of the liver. 4. Kidneys: Marked congestion and acute tubular necrosis. 5. Stomach: Congestion, hemorrhages, and mucosal necrosis. 6. Bite site: Hemorrhage, congestion, and dermal necrosis.	[126]

## Discussion

This systematic review and meta-analysis synthesized data from 130 cases of SBE associated with stroke, highlighting a rare but severe complication with high rates of mortality and long-term disability. Ischemic stroke was the predominant presentation, followed by hemorrhagic stroke and mixed forms. Most victims were male, of working age, and bitten during occupational or outdoor activities, circumstances that contribute to significant morbidity and socioeconomic impact. The principal genera involved were *Daboia*, *Bothrops*, and *Echis*, with *Daboia russelii* alone accounting for more than half of the reported cases. Stroke symptoms appeared within the first 24 hours in most cases, although delayed presentations up to seven days post-bite were also documented. Mortality was reported in 24% of cases, and among survivors, more than 70% experienced long-term sequelae, including motor deficits, speech impairments, cognitive alterations, and visual loss. These outcomes reinforce the need to consider stroke as a critical, disabling, and underrecognized outcome of SBE.

The demographic and clinical profiles identified in this review align with prior scoping reviews and observational studies, which also reported a predominance of ischemic events and a higher incidence among young adult males in rural or peri-urban settings [39]. These findings reflect the occupational exposure of this population, often involved in agricultural or manual labor, where protective measures are scarce and access to timely healthcare is limited. Similar to the results presented by Al-Sadawi et al. [134], the present review found that ischemic strokes occurred more frequently than hemorrhagic strokes. However, this distribution is likely underestimated, given the limitations in diagnostic imaging in many of the reported cases, particularly in low-resource settings. Additionally, consistent with the study by Mosquera et al. [135], most individuals affected did not have pre-existing cardiovascular risk factors, indicating a likely causal relationship between the envenomation and cerebrovascular insult, rather than an incidental association. Nonetheless, when comorbidities such as hypertension, diabetes, or nephropathies were present, they appeared to amplify the risk and severity of neurological injury [136].

The pathophysiological mechanisms underlying stroke following SBE are multifactorial and involve complex interactions between hemostatic disturbs, endothelial damage, and systemic hemodynamic alterations. The venom composition of different snake venoms is responsible for dictating different types of alterations, inducing or inhibiting cloting and platelet activation, presence of hemorrhagins and vascular damage [137,138]. In the present study, we identified hemostatic disturbances, particularly thrombocytopenia and venom-induced consumption coagulopathy (VICC), as frequent occurrences in both ischemic and hemorrhagic stroke cases - affecting over 60% and just under 50% of patients, respectively. Nevertheless, these alterations did not demonstrate sufficient discriminative power to differentiate between ischemic and hemorrhagic stroke subtypes. However, in the cases of mortality, thrombocytopenia demonstrated a significant association in fatal cases. Snake venoms, specially from Viperidae family, are known for its capacity to interfere in hemostasis. The action of venom proteases (metalo and serineproteases) is responsible for triggering clot formation by activation of different coagulation factors, inducing a procoagulant event, followed by a consumption of coagulation factors (VICC) and leading to a less coagulable status of the blood [139]. Regarding platelets, venom toxins are capable to alter platelet function, inducing a transient or persistent thrombocytopenia state, associated to bite site trauma activation, systemic thrombotic microangiopathy, or by organ-sequestration of platelets, and in most cases despite antivenom administration [10,140,141].

Another notable finding in the present study was that the development of sepsis emerged as a major determinant of mortality in SBE-related stroke, being associated with more than a sixfold increase in the likelihood of death. Sepsis raises stroke risk and can directly cause cerebral ischemia/hemorrhage through a combination of several disturbs such as systemic inflammation, endotheliopathy/blood–brain barrier disruption, coagulopathy with microthrombi, and impaired cerebral perfusion/autoregulation [142,143]. These pathological processes can amplify venom-induced disturbances, acting synergistically to events associated to coagulation-inflammation crosstalk, and endothelial injury, leading to increased vulnerability to ischemic or hemorrhagic stroke consequences [144,145].

The severity and clinical manifestations of stroke following SBE are strongly influenced by the species involved, the composition and quantity of venom injected, and host-related factors such as age, body mass, and number of bites. Variability in venom composition across snake populations – even within the same species – can lead to markedly different outcomes due to differing concentrations of procoagulant, hemorrhagic, and neurotoxic components [146]. This is particularly relevant in cases involving multiple bites or pediatric patients, where the venom dose-to-body-mass ratio is higher. A striking case described by Tibballs et al. [36] involved an 11-year-old child who suffered several bites to the wrist and rapidly developed fatal, extensive cerebral hemorrhages. Such reports emphasize that, beyond systemic coagulopathies, venom burden and host vulnerability are critical determinants of cerebrovascular complications. Additionally, species such as *D. russelii* and *Bothrops* spp. have been repeatedly implicated in cases of stroke, which may reflect both their biological potency and their epidemiological prevalence in certain regions. These associations underline the need for region-specific envenomation management protocols that consider the local snake fauna and potential for stroke-related sequelae.

Delayed access to medical care and antivenom therapy remains a major determinant of poor prognosis following SBE, particularly in low- and middle-income countries. In the Amazon region, for example, more than 70% of victims sought care only after 6 hours post-envenomation [14]. While several studies associate late administration of antivenom with increased risk of systemic complications – including stroke – our analysis identified multiple cases where stroke developed even after early access to antivenom therapy [14,15]. Some victims exhibited neurological deterioration within hours of the bite, with no apparent hypotension or vasculitis to explain the event [41,81], while others developed stroke several days later (typically 4–7 days), despite timely treatment [41,91].

Although our analysis did not demonstrate a significant association between delay in antivenom administration and the occurrence of stroke, the results suggest that secondary complications leading to death could be better mitigated through broader access to structured healthcare services. In many remote or low-resource areas, the lack of neuroimaging and intensive care support represents a major barrier to the timely recognition and management of neurological deterioration. Early identification of clinical warning signs - such as decreased level of consciousness, nausea, vomiting, or focal deficits - should therefore prompt rapid referral to facilities equipped with computed tomography and critical care resources. Establishing regional triage networks and strengthening community-level training for healthcare professionals may help reduce diagnostic delays. In parallel, incorporating telemedicine support and standardized stroke-alert protocols could enhance early detection and coordination of care. Considering the high fatality of hemorrhagic stroke, especially where imaging is unavailable, maintaining a high index of suspicion and ensuring early stabilization remain crucial to prevent avoidable deaths.

Also, an important consideration is the probable underreporting of stroke following snakebite envenoming. The true incidence, especially of ischemic events, is likely higher than documented, since many cases occur in regions without access to neuroimaging such as computed tomography or magnetic resonance. In these settings, neurological symptoms may be overlooked, misclassified as systemic manifestations, or never confirmed. Limited diagnostic capacity and delayed referrals further contribute to underestimation. Expanding access to imaging, improving training for early recognition of neurological signs, and strengthening surveillance systems are crucial to reveal the real burden of cerebrovascular complications caused by snakebite envenoming.

Despite the severity of neurological complications observed in many cases, most patients lacked structured follow-up after hospital discharge. Several factors contribute to this gap, including the long distances between patients’ homes and referral centers, limited transportation infrastructure in rural and forested regions, and the scarcity of specialized neurological and rehabilitation services. Consequently, the burden of post-stroke disabilities – such as motor impairment, cognitive deficits, and emotional disturbances – remains poorly documented and frequently unmanaged. Recent findings from studies on traumatic brain injury indicate that beyond motor and cognitive deficits, visual and sensory sequelae may also emerge due to diffuse cerebral damage, including inflammation, endothelial dysfunction, and disruption of the blood-brain barrier. These mechanisms, although more studied in trauma contexts, may be relevant in SBE-related strokes and could explain underrecognized symptoms such as visual disturbances or perceptual deficits [147] as found in this study. This under-recognition of long-term sequelae contributes to continued suffering, decreased quality of life, and socioeconomic instability, especially among working-age individuals who were envenomed during occupational activities. In this context, snakebite envenoming emerges not only as an acute medical emergency but also as a potential cause of long-term disability, requiring integration into broader strategies for non-communicable disease management and disability-inclusive health policies, particularly in endemic regions.

This study has some limitations. First, the retrospective design and reliance on secondary data may have led to information bias and missing variables, particularly in follow-up assessments. The heterogeneity in diagnostic methods (e.g., CT vs. MRI) and clinical documentation across cases may have influenced the classification of stroke subtypes and outcomes. In addition, the absence of a matched control group precludes direct causal inferences. Genetic predisposition, pre-existing vascular risk factors, and the specific composition of venoms (which can vary within species and across geography) could not be fully accounted for. Furthermore, sequelae were self-reported or clinically inferred, without standardized neurological or functional assessment tools, which limits their comparability in most cases.

## Conclusions

This systematic review and meta-analysis demonstrate that snakebite envenoming, while classically regarded as an acute medical emergency, can lead to severe neurological complications such as stroke, with high rates of mortality and long-term disability. These outcomes affect not only vulnerable populations with limited healthcare access but also previously healthy individuals, including those who received timely antivenom therapy. The lack of structured post-discharge care and access to rehabilitation services exacerbates the individual and societal burden of these events. Recognizing stroke as a disabling and underreported consequence of snakebite is essential for improving clinical outcomes and guiding public health responses. Efforts must be directed toward strengthening clinical surveillance, expanding diagnostic and rehabilitative capacity in endemic areas, and incorporating stroke-related outcomes into global snakebite burden estimates. Furthermore, investment in basic and translational research is needed to better understand the pathophysiological mechanisms involved, identify early predictors of neurological involvement, and develop species-specific antivenom formulations or adjunctive therapies. Integrating these insights into health policies will be vital for reducing long-term morbidity and advancing disability-inclusive strategies in neglected tropical disease programs.

## Supporting information

S1 File PRISMA checklist. PRISMA is licensed under a CCBY 4.0 license.
(DOCX)

S2 File Case reports of stroke secondary to snakebite envenomation. (DOCX)
