# Supplementary material for: Strokes following snakebite envenomations: A systematic review and individual patient data meta-analysis
Source: PLoS Negl Trop Dis. 2025 Dec 4;19(12):e0013789. doi: 10.1371/journal.pntd.0013789 (PMC12677522; doi:10.1371/journal.pntd.0013789)
Supplement: S2 File — (DOCX) [file pntd.0013789.s002.docx]

# Supplementary table 1. Case reports of stroke secondary to snakebite envenomation

| **First author** | **Year** | **Country** | **Age** | **Gender** | **Snake** | **Type** | **Stroke type** | **Diagnostic method** | **Treatment** | **Outcome** |
| --- | --- | --- | --- | --- | --- | --- | --- | --- | --- | --- |
| Zeng X | 2019 | China | 49 | F | *T. Stejnegeri* | Ischemic | Posterior cerebral artery infarction | Brain computed tomography and magnetic resonance imaging | Conservative | Partial recovery |
| Silva De Oliveira | 2017 | Brazil | 59 | F | *Bothrops atrox* | Hemorrhagic | Not available / Not specified | Imaging method not reported / unavailable | Conservative | Death |
| Lahiri D | 2019 | India | 50 | M | *Viper* | Ischemic | Middle cerebral artery infarction | Brain computed tomography and magnetic resonance imaging | Conservative | Partial recovery |
| Sachett J. | 2020 | Brazil | 65 | F | *Bothrops atrox* | Hemorrhagic | Multiple intracerebral hemorrhages | Brain computed tomography (CT) | Conservative | Complete recovery |
| Sachett J. | 2020 | Brazil | 22 | M | *Jararaca* | Hemorrhagic | Multiple intracerebral hemorrhages | Brain computed tomography (CT) | Conservative | Death |
| Pérez-Gómez As | 2020 | Brazil | 15 | M | *Bothrops* | Hemorrhagic | Anterior cerebral artery infarction | Brain computed tomography (CT) | Conservative | Complete recovery |
| Pérez-Gómez As | 2019 | Brazil | 78 | F | *Bothrops* | Hemorrhagic | Middle cerebral artery infarction | Brain computed tomography (CT) | Conservative | Partial recovery |
| Pérez-Gómez As | 2019 | Brazil | 20 | F | *Bothrops* | Hemorrhagic | Multiple intracerebral hemorrhages | Brain computed tomography (CT) | Conservative | Partial recovery |
| Zhang T | 2018 | China | 34 | M | *Deinagkistrodon aculus* | Ischemic | Multiple cerebral infarctions | Brain computed tomography (CT) | Conservative | Death |
| Delgado Abt | 2017 | Brazil | 58 | F | *Bothrops Spp* | Hemorrhagic | Multiple intracerebral hemorrhages | Brain computed tomography (CT) | Conservative | Partial recovery |
| Ittyachen Am | 2012 | India | 55 | M | *Russell viper* | Ischemic | Posterior cerebral artery infarction | Brain magnetic resonance imaging (MRI) | Conservative | Complete recovery |
| Bush Sp | 2014 | United States | 50 | M | *Crotalus oreganus helleri* | Ischemic | Multiple intracerebral hemorrhages | Brain computed tomography and magnetic resonance imaging | Conservative | Death |
| Bush Sp | 2014 | United States | 17 | M | *Crotalus oreganus helleri* | Ischemic | Middle cerebral artery infarction | Brain magnetic resonance imaging (MRI) | Conservative | Partial recovery |
| Namal Rathnayaka Rm | 2017 | Sri lanka | 53 | M | *Russell viper* | Hemorrhagic | Multiple infarctions with secondary hemorrhagic transformation | Brain computed tomography (CT) | Conservative | Death |
| Narang Sk | 2009 | India | 18 | M | *Russell Viper* | Ischemic | Middle cerebral artery infarction | Brain computed tomography and magnetic resonance imaging | Conservative | Partial recovery |
| Silveira Gg | 2016 | Brazil | 52 | M | *Bothrops jararacussu* | Hemorrhagic | Multiple intracerebral hemorrhages | Brain computed tomography (CT) | Conservative | Complete recovery |
| Santos-Soares Pc | 2007 | Brazil | 65 | F | *Jararaca* | Hemorrhagic | Multiple intracerebral hemorrhages | Brain computed tomography (CT) | Surgical | Complete recovery |
| Cañas Ca | 2016 | Colombia | 48 | F | *Bothrops atrox* | Ischemic | Multiple cerebral infarctions | Brain magnetic resonance imaging (MRI) | Conservative | Partial recovery |
| Rebahi H | 2014 | Morocco | 32 | F | *Cerastes cerastes* | Ischemic | Multiple cerebral infarctions | Brain computed tomography (CT) | Conservative | Death |
| Rebahi H | 2014 | Morocco | 5 | F | *Cerastes cerastes* | Ischemic | Multiple cerebral infarctions | Brain computed tomography (CT) | Conservative | Death |
| Rebahi H | 2014 | Morocco | 51 | M | *Cerastes cerastes* | Ischemic | Multiple cerebral infarctions | Brain computed tomography (CT) | Conservative | Complete recovery |
| Chani M | 2012 | Morocco | 55 | M | *Cerastes cerastes* | Ischemic | Multiple infarctions with secondary hemorrhagic transformation | Brain computed tomography (CT) | Conservative | Complete recovery |
| Vale Tc | 2013 | Brazil | 16 | M | *Crotalus durissis Terrificus* | Ischemic | Multiple cerebral infarctions | Brain computed tomography and magnetic resonance imaging | Conservative | Partial recovery |
| Pothukuchi Vk | 2017 | India | 70 | M | *Russel viper* | Ischemic | Middle cerebral artery infarction | Brain computed tomography (CT) | Conservative | Complete recovery |
| Pothukuchi Vk | 2017 | India | 55 | M | *Russel viper* | Ischemic | Anterior cerebral artery infarction | Brain computed tomography (CT) | Conservative | Complete recovery |
| Gawarammana I | 2009 | Sri Lanka | 56 | M | *Daboia russelli* | Ischemic | Multiple cerebral infarctions | Brain computed tomography (CT) | Conservative | Complete recovery |
| Gawarammana I | 2009 | Sri Lanka | 37 | M | *Daboia russelli* | Ischemic | Middle cerebral artery infarction | Brain computed tomography (CT) | Conservative | Complete recovery |
| Gawarammana I | 2009 | Sri Lanka | 45 | F | *Daboia russelli* | Ischemic | Multiple cerebral infarctions | Brain computed tomography (CT) | Conservative | Complete recovery |
| Gawarammana I | 2009 | Sri Lanka | 45 | F | *Daboia russelli* | Ischemic | Multiple cerebral infarctions | Brain computed tomography (CT) | Conservative | Partial recovery |
| Gawarammana I | 2009 | Sri Lanka | 28 | M | *Daboia russelli* | Ischemic | Multiple cerebral infarctions | Brain computed tomography (CT) | Conservative | Death |
| Gawarammana I | 2009 | Sri Lanka | 53 | M | *Daboia russelli* | Ischemic | Multiple cerebral infarctions | Brain computed tomography (CT) | Conservative | Partial recovery |
| Gawarammana I | 2009 | Sri Lanka | 35 | M | *Daboia russelli* | Ischemic | Middle cerebral artery infarction | Brain computed tomography (CT) | Conservative | Partial recovery |
| Gawarammana I | 2009 | Sri Lanka | 39 | M | *Daboia russelli* | Ischemic | Multiple cerebral infarctions | Brain computed tomography (CT) | Conservative | Partial recovery |
| Gawarammana I | 2009 | Sri Lanka | 54 | M | *Daboia russelli* | Ischemic | Middle cerebral artery infarction | Brain computed tomography (CT) | Conservative | Complete recovery |
| Bhatt A | 2013 | India | 65 | M | *Russel viper* | Ischemic | Multiple cerebral infarctions | Brain magnetic resonance imaging (MRI) | Conservative | Partial recovery |
| Thomas L | 2006 | France | 46 | M | *Bothrops lanceolatus* | Ischemic | Multiple cerebral infarctions | Brain magnetic resonance imaging (MRI) | Conservative | Complete recovery |
| Thomas L | 2006 | France | 55 | M | *Bothrops lanceolatus* | Ischemic | Middle cerebral artery infarction | Brain magnetic resonance imaging (MRI) | Conservative | Partial recovery |
| Thomas L | 2006 | France | 66 | M | *Bothrops lanceolatus* | Ischemic | Multiple cerebral infarctions | Brain magnetic resonance imaging (MRI) | Conservative | Partial recovery |
| Aissaoui Y | 2013 | Morocco | 72 | M | *Cerastes cerastes* | Hemorrhagic | Multiple infarctions with secondary hemorrhagic transformation | Brain computed tomography (CT) | Conservative | Complete recovery |
| Boviatsis Ej | 2003 | Greece | 65 | F | *Viper* | Hemorrhagic | Multiple intracerebral hemorrhages | Brain computed tomography (CT) | Conservative | Partial recovery |
| Aravanis C | 1982 | Greece | 17 | F | *Echis carinatus* | Ischemic | Not available / Not specified | Imaging method not reported / unavailable | Conservative | Partial recovery |
| Machado As | 2010 | Brazil | 62 | F | *Bothrops* | Hemorrhagic | Multiple intracerebral hemorrhages | Brain computed tomography (CT) | Conservative | Complete recovery |
| Mugundhan K | 2008 | India | 14 | M | Not reported | Ischemic | Multiple cerebral infarctions | Brain computed tomography (CT) | Conservative | Death |
| Hoskote Ss | 2009 | India | 24 | M | *Viper* | Ischemic | Multiple cerebral infarctions | Brain magnetic resonance imaging (MRI) | Conservative | Partial recovery |
| Bashir R | 1985 | Saudi Arabia | 13 | F | *Echis Carinatus* | Ischemic | Middle cerebral artery infarction | Brain computed tomography (CT) | Conservative | Partial recovery |
| Sahoo Ak | 2018 | India | 36 | M | *Viper* | Ischemic | Middle cerebral artery infarction | Imaging method not reported / unavailable | Conservative | Complete recovery |
| Smith H | 2019 | Australia | 2 | M | *Pseudonaja textili* | Ischemic | Multiple cerebral infarctions | Brain magnetic resonance imaging (MRI) | Conservative | Complete recovery |
| Jeevagan V | 2012 | Sri lanka | 65 | M | *Hypnale hypnale* | Ischemic | Middle cerebral artery infarction | Brain computed tomography (CT) | Conservative | Partial recovery |
| Gouda S | 2011 | India | 40 | F | *Russell viper* | Ischemic | Basilar artery infarction | Brain computed tomography and magnetic resonance imaging | Conservative | Partial recovery |
| De Oliveira Pardal Pp | 2015 | Brazil | 10 | M | *B. marajoensis* | Hemorrhagic | Anterior cerebral artery infarction | Imaging method not reported / unavailable | Conservative | Partial recovery |
| Lee | 2001 | Republic of Korea | 54 | M | *Agkistrodon blomhoffii brevicaudus* | Ischemic | Basilar artery infarction | Brain computed tomography (CT) | Conservative | Partial recovery |
| Tibbals | 1991 | Australia | 11 | M | *Notechis scutatus* | Hemorrhagic | Multiple intracerebral hemorrhages | Brain computed tomography (CT) | Conservative | Death |
| Menon | 2017 | India | 28 | M | *Viper* | Hemorrhagic | Anterior cerebral artery infarction | Brain computed tomography (CT) | Surgical | Complete recovery |
| Hsaini | 2010 | France | 24 | M | *Cerastes cerastes* | Ischemic | Multiple cerebral infarctions | Brain computed tomography (CT) | Conservative | Partial recovery |
| Ghezala Hb | 2015 | Tunisia | 37 | M | *Viper* | Hemorrhagic | Multiple infarctions with secondary hemorrhagic transformation | Brain magnetic resonance imaging (MRI) | Conservative | Death |
| Cole M | 1996 | United States | 43 | M | *Crotalus viridis viridis* | Ischemic | Middle cerebral artery infarction | Brain magnetic resonance imaging (MRI) | Conservative | Complete recovery |
| Diaz J | 2003 | Colombia | 11 | M | *Bothrops* | Ischemic | Multiple cerebral infarctions | Brain magnetic resonance imaging (MRI) | Conservative | Partial recovery |
| Viana Dc | 2009 | Brazil | 62 | F | *Bothrops Spp* | Hemorrhagic | Multiple intracerebral hemorrhages | Brain computed tomography (CT) | Conservative | Partial recovery |
| Deepu | 2011 | India | 48 | F | *Daboia russelli* | Ischemic | Basilar artery infarction | Brain computed tomography (CT) | Surgical | Partial recovery |
| Jalal M | 2016 | India | 48 | M | Not reported | Hemorrhagic | Multiple intracerebral hemorrhages | Brain computed tomography (CT) | Surgical | Complete recovery |
| Pinho F | 2009 | Brazil | 64 | F | *Bothrops jararacussu* | Hemorrhagic | Not available / Not specified | Imaging method not reported / unavailable | Conservative | Death |
| Chandrashekar A | 2014 | India | 40 | F | *Russell viper* | Ischemic | Middle cerebral artery infarction | Brain magnetic resonance imaging (MRI) | Conservative | Partial recovery |
| Benjamin J | 2019 | Benin | 15 | F | *Echis ocellatus* | Hemorrhagic | Not available / Not specified | Imaging method not reported / unavailable | Conservative | Complete recovery |
| Benjamin J | 2019 | Benin | 18 | M | *Echis ocellatus* | Hemorrhagic | Not available / Not specified | Imaging method not reported / unavailable | Conservative | Complete recovery |
| Lizarazo J | 2020 | Colombia | 59 | M | *Bothrops asper* | Hemorrhagic | Multiple intracerebral hemorrhages | Brain computed tomography (CT) | Surgical | Death |
| Bhojarara M | 2016 | India | 45 | F | Not reported | Ischemic | Multiple cerebral infarctions | Brain magnetic resonance imaging (MRI) | Conservative | Partial recovery |
| Gopalan S | 2014 | India | 32 | F | *Russell viper* | Ischemic | Multiple cerebral infarctions | Brain computed tomography (CT) | Conservative | Partial recovery |
| Ajit D | 2013 | India | 30 | F | *Viper* | Ischemic | Middle cerebral artery infarction | Brain computed tomography (CT) | Conservative | Partial recovery |
| Janardanaaithala R | 2017 | India | 38 | F | *Russell viper* | Ischemic | Multiple cerebral infarctions | Brain computed tomography (CT) | Conservative | Partial recovery |
| Jeyaraj M | 2016 | India | 25 | F | *Viper* | Ischemic | Multiple cerebral infarctions | Brain computed tomography and magnetic resonance imaging | Conservative | Complete recovery |
| Sahoo Lk | 2021 | India | 36 | M | *Daboia russelli* | Ischemic | Multiple cerebral infarctions | Brain computed tomography (CT) | Conservative | Partial recovery |
| Paul R | 2016 | India | 75 | M | *Daboia russelli* | Ischemic | Multiple cerebral infarctions | Brain computed tomography (CT) | Conservative | Complete recovery |
| Gupta S | 2012 | India | 48 | F | *Daboia russelli* | Ischemic | Basilar artery infarction | Brain computed tomography (CT) | Surgical | Partial recovery |
| Hung Dz | 2002 | Taiwan | 67 | M | *Daboia russelli* | Ischemic | Multiple cerebral infarctions | Brain computed tomography (CT) | Conservative | Partial recovery |
| Hung Dz | 2002 | Taiwan | 52 | F | *Daboia russelli* | Ischemic | Multiple cerebral infarctions | Brain computed tomography (CT) | Conservative | Death |
| Midyett Fa | 1998 | Australia | 57 | F | *Pseudonaja textili* | Hemorrhagic | Multiple intracerebral hemorrhages | Brain computed tomography (CT) | Conservative | Death |
| Subasinghe C | 2014 | Sri lanka | 54 | F | *Daboia russelli* | Ischemic | Posterior cerebral artery infarction | Brain computed tomography (CT) | Conservative | Partial recovery |
| Merle H | 2005 | Martinique | 46 | M | *Bothrops lanceolatus* | Ischemic | Posterior cerebral artery infarction | Brain magnetic resonance imaging (MRI) | Conservative | Partial recovery |
| Belhachmi | 2021 | Morocco | 62 | F | *Cerastes cerastes* | Hemorrhagic | Multiple intracerebral hemorrhages | Brain computed tomography (CT) | Conservative | Death |
| Kumar R | 2015 | India | 32 | M | *Daboia russelli* | Ischemic | Multiple cerebral infarctions | Brain magnetic resonance imaging (MRI) | Conservative | Partial recovery |
| Numeric P | 2002 | Martinique | 32 | M | *Bothrops lanceolatus* | Ischemic | Multiple cerebral infarctions | Brain magnetic resonance imaging (MRI) | Conservative | Partial recovery |
| Pal J | 2014 | India | 21 | M | *Viper* | Ischemic | Middle cerebral artery infarction | Brain computed tomography (CT) | Conservative | Partial recovery |
| Kodiatte Abraham A | 2019 | India | 33 | M | *Viper* | Ischemic | Multiple cerebral infarctions | Imaging method not reported / unavailable | Conservative | Partial recovery |
| Sk Das | 2013 | India | 27 | F | *Daboia russelli* | Ischemic | Middle cerebral artery infarction | Brain magnetic resonance imaging (MRI) | Conservative | Complete recovery |
| Malbranque S | 2008 | Martinique | 74 | M | *Bothrops lanceolatus* | Ischemic | Multiple cerebral infarctions | Brain magnetic resonance imaging (MRI) | Conservative | Death |
| Kitchens C | 2008 | United States | 54 | M | *Crotalus adamanteus* | Hemorrhagic | Multiple intracerebral hemorrhages | Brain computed tomography (CT) | Conservative | Death |
| Fonseka C | 2013 | Sri Lanka | 19 | M | *Echis carinatus* | Hemorrhagic | Multiple intracerebral hemorrhages | Brain computed tomography (CT) | Conservative | Complete recovery |
| Pinzon R | 2022 | Indonesia | 72 | M | *Calloselasma rhodostoma* | Ischemic | Anterior cerebral artery infarction | Brain computed tomography (CT) | Conservative | Complete recovery |
| Krishna P | 2017 | India | 30 | F | Not reported | Ischemic | Middle cerebral artery infarction | Brain computed tomography (CT) | Conservative | Complete recovery |
| Dabilgou A | 2021 | Burkina Faso | 55 | F | Not reported | Hemorrhagic | Middle cerebral artery infarction | Brain computed tomography (CT) | Conservative | Partial recovery |
| Dabilgou A | 2021 | Burkina Faso | 16 | M | Not reported | Hemorrhagic | Posterior cerebral artery infarction | Brain computed tomography (CT) | Conservative | Complete recovery |
| Dabilgou A | 2021 | Burkina Faso | 30 | F | Not reported | Hemorrhagic | Multiple intracerebral hemorrhages | Brain computed tomography (CT) | Conservative | Complete recovery |
| Parasher A | 2020 | India | 64 | M | Not reported | Hemorrhagic | Anterior cerebral artery infarction | Brain computed tomography (CT) | Conservative | Partial recovery |
| Goswami S | 2019 | India | 75 | M | Not reported | Hemorrhagic | Multiple intracerebral hemorrhages | Brain computed tomography (CT) | Conservative | Partial recovery |
| Sathishkumar J | 2017 | India | 45 | M | Not reported | Ischemic | Middle cerebral artery infarction | Brain computed tomography (CT) | Conservative | Partial recovery |
| Kumar N | 2014 | India | 22 | M | *Echis carinatus* | Hemorrhagic | Middle cerebral artery infarction | Brain computed tomography (CT) | Conservative | Complete recovery |
| Mahale R | 2014 | India | 58 | M | *Trimeresurus gramineus* | Ischemic | Multiple cerebral infarctions | Brain computed tomography and magnetic resonance imaging | Conservative | Partial recovery |
| Singh S | 1997 | India | 23 | M | Not reported | Hemorrhagic | Multiple infarctions with secondary hemorrhagic transformation | Brain computed tomography (CT) | Conservative | Death |
| Paul G | 2014 | India | 36 | M | Not reported | Hemorrhagic | Basilar artery infarction | Brain magnetic resonance imaging (MRI) | Conservative | Complete recovery |
| Paul G | 2014 | India | 40 | M | Not reported | Hemorrhagic | Middle cerebral artery infarction | Brain magnetic resonance imaging (MRI) | Conservative | Partial recovery |
| Ghosh R | 2022 | India | 40 | F | *Daboia russellii* | Ischemic | Not available / Not specified | Brain magnetic resonance imaging (MRI) | Conservative | Complete recovery |
| Rmmk Nr | 2022 | Sri Lanka | 71 | M | *Hypnale hypnale* | Ischemic | Middle cerebral artery infarction | Brain computed tomography (CT) | Conservative | Complete recovery |
| Debajyoti D | 2021 | India | 62 | M | Not reported | Hemorrhagic | Middle cerebral artery infarction | Brain computed tomography (CT) | Surgical | Complete recovery |
| Iwuozo Eu | 2022 | Nigeria | 18 | F | Not reported | Hemorrhagic | Multiple intracerebral hemorrhages | Brain computed tomography (CT) | Conservative | Partial recovery |
| Hawa Ka | 2022 | Morocco | 56 | M | *Viper* | Hemorrhagic | Multiple intracerebral hemorrhages | Brain computed tomography (CT) | Conservative | Death |
| Mouhssine A | 2022 | Morocco | 6 | F | *Cerastes cerastes* | Ischemic | Multiple infarctions with secondary hemorrhagic transformation | Brain computed tomography (CT) | Conservative | Death |
| Yalcouyé A | 2021 | Mali | 6 | M | Not reported | Hemorrhagic | Anterior cerebral artery infarction | Brain computed tomography (CT) | Conservative | Partial recovery |
| Ouedraogo Pv | 2022 | Burkina Faso | 60 | F | *Viper* | Hemorrhagic | Multiple intracerebral hemorrhages | Brain computed tomography (CT) | Conservative | Partial recovery |
| Ouedraogo Pv | 2022 | Burkina Faso | 50 | F | *Viper* | Hemorrhagic | Multiple intracerebral hemorrhages | Brain computed tomography (CT) | Conservative | Partial recovery |
| Villota Vam | 2022 | Colombia | 50 | M | *Bothrops spp.* | Ischemic | Multiple cerebral infarctions | Brain computed tomography and magnetic resonance imaging | Conservative | Partial recovery |
| Yahaya, Snb | 2023 | Malaysia | 58 | M | Not reported | Ischemic | Multiple cerebral infarctions | Brain computed tomography (CT) | Conservative | Death |
| Sachett Jag | 2024 | Brazil | 52 | M | *Bothrops atrox* | Hemorrhagic | Multiple intracerebral hemorrhages | Brain computed tomography (CT) | Conservative | Death |
| Mouad L | 2024 | Morocco | 15 | M | Not reported | Ischemic | Multiple infarctions with secondary hemorrhagic transformation | Brain computed tomography (CT) | Conservative | Death |
| Freston Ms | 2022 | India | 60 | F | *Hypnale hypnale* | Hemorrhagic | Basilar artery infarction | Brain computed tomography (CT) | Conservative | Death |
| Freston Ms | 2022 | India | 25 | M | *Hypnale hypnale* | Hemorrhagic | Middle cerebral artery infarction | Brain computed tomography (CT) | Conservative | Outcome not reported / unavailable |
| Sharath P | 2023 | India | 26 | M | Not reported | Ischemic | Multiple cerebral infarctions | Brain computed tomography (CT) | Conservative | Complete recovery |
| Reddy Sn | 2023 | India | 28 | M | Not reported | Ischemic | Multiple cerebral infarctions | Brain magnetic resonance imaging (MRI) | Conservative | Outcome not reported / unavailable |
| Nakipuria M | 2023 | India | 50 | F | Not reported | Ischemic | Posterior cerebral artery infarction | Brain computed tomography and magnetic resonance imaging | Conservative | Death |
| Nakipuria M | 2023 | India | 37 | F | Not reported | Ischemic | Basilar artery infarction | Brain computed tomography (CT) | Surgical | Complete recovery |
| Nakipuria M | 2023 | India | 54 | M | Not reported | Ischemic | Multiple cerebral infarctions | Brain magnetic resonance imaging (MRI) | Treatment not reported / unavailable | Partial recovery |
| Nakipuria M | 2023 | India | 58 | F | Not reported | Ischemic | Multiple cerebral infarctions | Brain magnetic resonance imaging (MRI) | Conservative | Partial recovery |
| Nakipuria M | 2023 | India | 55 | F | Not reported | Ischemic | Multiple cerebral infarctions | Brain magnetic resonance imaging (MRI) | Conservative | Death |
| Sahu Mr | 2023 | India | 42 | M | Not reported | Hemorrhagic | Multiple intracerebral hemorrhages | Brain computed tomography (CT) | Conservative | Death |
| Das Ds | 2024 | India | 30 | F | Not reported | Ischemic | Posterior cerebral artery infarction | Brain computed tomography and magnetic resonance imaging | Conservative | Complete recovery |
| Bentes Ko | 2024 | Brazil | 54 | F | Not reported | Ischemic | Middle cerebral artery infarction | Brain computed tomography (CT) | Conservative | Partial recovery |
| Kouyoumdjian Aj | 1989 | Brazil | 13 | M | *Bothrops moojeni* | Hemorrhagic | Anterior cerebral artery infarction | Brain computed tomography (CT) | Conservative | Death |
| Kenny Mejías Md | 2024 | Venezuela | 13 | F | *Bothrops Sp* | Hemorrhagic | Middle cerebral artery infarction | Brain computed tomography (CT) | Surgical | Partial recovery |
| Dellandrea H | 2024 | Brazil | 11 | M | *Bothrops Sp* | Hemorrhagic | Multiple intracerebral hemorrhages | Brain computed tomography (CT) | Conservative | Death |
| Florentin J | 2024 | France | 93 | M | *Bothrops lanceolatus* | Ischemic | Multiple cerebral infarctions | Brain computed tomography and magnetic resonance imaging | Conservative | Complete recovery |
| Florentin J | 2024 | France | 84 | M | Not reported | Hemorrhagic | Multiple intracerebral hemorrhages | Brain computed tomography and magnetic resonance imaging | Conservative | Complete recovery |
